# CXCR4 Based Therapeutics for Non-Small Cell Lung Cancer (NSCLC)

**DOI:** 10.3390/jcm7100303

**Published:** 2018-09-25

**Authors:** Ori Wald

**Affiliations:** 1 Department of Cardiothoracic Surgery, Hadassah Hebrew University Hospital, Jerusalem 91120, Israel; ori.wald@mail.huji.ac.il; Tel.: +972-50-894-6297; Fax: +972-2-534-5125; 2 Goldyne Savad Institute of Gene Therapy, Hadassah Hebrew University Hospital, Jerusalem 91120, Israel

**Keywords:** NSCLC, lung, chemokines, immune checkpoint inhibitors

## Abstract

Lung cancer is the second most common malignancy. Unfortunately, despite advances in multimodality therapeutics for the disease, the overall five-year survival rate among newly diagnosed lung cancer patients remains in the range region of 15%. In addition, although immune checkpoint inhibitors are increasingly being incorporated into lung cancer treatment protocols, the proportion of patients that respond to these agents remains low and the duration of response is often short. Therefore, novel methodologies to enhance the efficacy of immunotherapy in lung cancer are highly desirable. Chemokines are small chemotactic cytokines that interact with their 7 transmembrane G-protein–coupled receptors, to guide immune cell trafficking in the body under both physiologic and pathologic conditions. Tumor cells highjack a small repertoire of the chemokine/chemokine receptor system and utilize it in a manner that benefits local tumor growth and distant spread. The chemokine receptor, CXCR4 is expressed in over 30 types of malignant tumors and, through interaction with its ligand CXCL12, was shown exert pleotropic pro-tumorigenic effects. In this review, the pathologic roles that CXCL12/CXCR4 play in lung cancer propagation are presented. Furthermore, the challenges and potential benefits of incorporating drugs that target CXCL12/CXCR4 into immune-based lung cancer therapeutic protocols are discussed.

## 1. Lung Cancer Epidemiology and Classification

Lung cancer is the second most common malignancy and the leading cause of cancer-related death among both men and women. Indeed, it is estimated that 25% of all cancer-related mortality is due to lung cancer [1]. Environmental and genetic factors influence lung cancer pathogenesis with cigarette smoke being the major environmental risk factor and a growing number of genetic alterations (including activating mutations in EGFR—Epidermal growth factor receptor, cMET—Hepatocyte growth factor receptor transmembrane tyrosine kinase pathway, ALK—Anaplastic lymphoma receptor tyrosine kinase and ROS-1—ROS proto-oncogene 1, receptor tyrosine kinase) being recognized as the driving forces underlying lung cancer development in non-smokers [2,3]. In 2018, over 230,000 new cases of lung cancer will be diagnosed in the United States alone and over 150,000 deaths attributed to the disease. Changes in smoking behavior over the past decades have resulted in a decline in the incidence of lung cancer among men (current lung cancer incidence rate of 55 per 100,000). In contrast, changes in behavior (increased consumption of tobacco in the earlier year) first led to a rise and then to a stabilization of lung cancer incidence among women (current lung cancer incidence rate of 40 per 100,000) [1]. Remarkably, a trend of increased frequency of lung cancer among non-smoking young women has recently been reported, though the causes of this phenomena are yet to be clarified [4]. Despite advances in lung cancer screening and diagnosis, the majority of lung cancer cases are still diagnosed relatively late in the course of disease and the overall five-year lung cancer related mortality rate remains high (around 80%). Thus, lung cancer related morbidity and mortality remain a major global health problem and novel therapeutics for the disease are highly desirable [5]. 

In 2015, the World Health Organization (WHO) revised the classifications of tumors of the lung. It defined three major types of malignant epithelial tumors of the lung namely adenocarcinomas, squamous cell carcinomas and neuroendocrine tumors of the lung. The term non-small cell lung cancer (NSCLC) refers to lung adeno and squamous carcinomas and accounts for nearly 80% of all newly diagnosed lung cancer cases, whereas the term small cell lung cancer (SCLC) refers to the most malignant neuroendocrine tumor of the lung and accounts for the remaining 20%. As their name indicates, large cell neouroendocire carcinomas (around 2% of cases) belong to the neuroendocrine subtype of tumors; they are considered as aggressive as SCLC [6,7,8]. Lung adenocarcinoma is the most common histologic subtype in most countries, accounting for almost half of all lung cancers. The histopathological, radiological and molecular spectrum of lung adenocarcinoma is highly diverse with some tumors showing a very benign clinical course and others being highly aggressive [9,10]. The majority of data reviewed in this manuscript relate to lung adenocarcinomas.

## 2. Lung Cancer Staging and Treatment

Similar to other solid malignancies, lung cancer is staged according to the TNM classification which determines three key parameters (T–the extent of the primary tumor, N–the presence or absence of lymph nodes metastases, M-the presence or absence of distant metastases) that together measure the local and systemic extent of the disease [11]. Clinical disease staging is initially achieved by imaging modalities such as computed tomography scans (CT), positron emission tomography CT scans (PET-CT) and magnetic resonance imaging (MRI). In equivocal cases, these scans are complemented by more invasive tests, such as endo-bronchial ultrasound (EBUS) and mediastinoscopy. Disease stage guides the preferred therapeutic intervention with early stage disease (Stage IA-IIB) being treated primarily by surgery and more advanced disease stages (IIIB-IVB) being treated with chemotherapy, radiotherapy and biological treatments as dictated by genetic analysis of the tumor. Stage IIIA denotes a locally advanced disease that is often treated using a multimodality approach that combines chemotherapy, radiotherapy and biological treatments with surgery, to achieve extended survival [2,12,13] (see also NCCN—National Comprehensive Cancer Network—guidelines for management of NSCLC).

In recent years, Immune Checkpoint Inhibitors (ICI) have revolutionized the field of cancer therapeutics emerging as key drugs in the combat of a growing number of malignancies [14]. In the context of lung cancer, research has also studied their effect in NSCLC patients presenting with advanced disease which is not amenable to treatment with biological agents (i.e., tumors which do not carry a targetable genetic alternation that can be addressed with specific drugs such as EGFR and ALK inhibitors). It was shown that when ICIs were added to conventional chemotherapeutic protocols, promising life-extending effects were observed [15]. Consequently, ICIs are being adopted as the first line of therapeutic options for biologically-untargetable, advanced NSCLC [16]. In addition, studies are also planned to evaluate the efficacy of ICIs as neo-adjuvant treatments in the setting of early-stage surgically treatable NSCLC. In this scenario, researchers are looking both at the short-term effects of ICI on the tumor viability (as assessed by histological analysis of the resected tumor), and at the long-term effects of ICIs on disease recurrence rates [17]. However, as promising as ICIs may seem for disease treatment, it is important to recall the following: (a) the overall response rates to ICI in NSCLC remain relatively low (15–50%); (b) the duration of response to ICIs is relatively limited (12 months) with “cure” remaining an unmet goal; and (c) the side effect profile of ICIs is significant and consequently many patients—especially those who are being treated with more than one ICI—withdraw treatment due to its associated autoimmune symptoms [18,19,20]. 

Several clinical biomarkers including the tumor mutational burden (as determined by next generation sequencing), immunohistochemistry staining for PD-L1, and effector T-cell gene signature have been developed to identify NSCLC patients that may better respond to ICI. However, with the broader objective being expanding the population of patients that may benefit from ICI, it is important to explore novel methodologies/therapeutics that would increase the proportion of patients that respond to such treatments while simultaneously avoiding augmentation of their side effect profile [21,22]. This review discusses the antagonism of the chemokine CXCL12 and its receptor CXCR4 as one such potential approach.

## 3. Chemokine and Chemokine Receptors in Solid Malignancies

Chemokines (CK) are small tissue produced chemotactic cytokines. They signal via their receptors (a subclass of the seven transmembrane G-protein–coupled receptors—chemokine receptors—CKR) that are expressed by immune cells to coordinate leukocyte trafficking under both physiologic and pathologic conditions [23]. Chemokines are classified according to their structure not function. Four subdivisions are named according to the number and spacing of conserved N-terminal cysteine residues. According to this nomenclature, C denotes the cysteine residue and X denotes number of amino acids that are spaced between them (CC—no spacing, CXC—spacing of one residue and CX3C—spacing of three residues). All but three chemokine are in the CXC and CC group. Altogether, there are 28 CC chemokine (termed CCL1 to CCL28) and 17 CXC chemokines (termed CXCL1 to CXCL17). Correspondingly, there are 10 CC receptors (termed CCR1 to CCR10) and 6 CXC receptors (termed CXCR1 to CXCR6). In addition, there are six atypical chemokine (ACKR) receptors. Distinct from the typical chemokine receptors (CKR), these ACKR do not signal via the Gi unit but rather act to shape chemokine gradients and dampen inflammation by scavenging chemokines in a G protein-independent but rather betta-arrestin-dependent manner [24]. In addition to immune cells, tumor cells are also known to express CKs and CKRs. Although they express a relatively restricted repertoire of CKs and CKRs, they efficiently manipulate this system to support their local growth and distant spread. In particular, in the tumor microenvironment (TME), autocrine and paracrine chemokine/chemokine receptor loops interact to promote tumor cell survival and growth, and also to enhance tumor neo-angiogenesis [25,26]. In addition, tumor produced chemokines attract immune-suppressive cells into the tumors and thus inhibit the generation of an effective anti-tumor immune response. At distant sites, it is the tissue-produced chemokine which guides/attracts the metastasis of chemokine-receptor-expressing tumor cells to the tissue [27].

## 4. CXCL12, CXCR4 and ACKR3 (CXCR7) in Solid Malignancies

The majority of CKs activate more than one CKR and most CKRs bind more than one CK. However, unique to this system are the monogamous CK/CKR pair CXCL12/CXCR4 and their associated scavenger receptor ACKR3 (also known as CXCR7) [24]. The CXCL12/CXCR4/ACKR3 axis performs multi-system multitasking during embryogenesis as well as during homeostasis and at sites of inflammation/infection. The central evolutionary role of this axis is highlighted by the fact that Distinct from all other CK/CKRs inactivating mutations, this triad of proteins has been associated with fatal embryonic defects. Moreover, activating mutation in this axis is associated with a specific genetically driven immunologic deficiency. Key roles for this axis have been demonstrated in hematopoiesis, in cardiac and cerebellar organogenesis, and in embryonal peripheral nerve guidance. Further, non-lethal activating mutations in the CXCR4 receptor are known to cause the WHIM syndrome, a syndrome manifested by Warts, Hypogammaglobulinemia, recurrent Infections, and Myelokathexis (a term used to describe bone marrow retention–mainly of neutrophils) [28]. The CXCL12/CXCR4/ACKR3 axis is frequently hijacked by cancer cells to promote their survival, growth, resistance to chemotherapy, metastasis and immune evasion [27,29]. In particular, activation of various oncological pathways, such as (a) the RET/PTC rearrangement in papillary thyroid carcinomas [30], (b) the human epithelial growth factor receptor HER-2 in breast cancer cells [31], (c) the Hypoxia inducible factor 1 (HIF-1), and (d) the vascular endothelial growth factor (VEGF) in glioblastoma [32] have all been shown to drive CXCR4 over-expression in cancer cells making them highly responsive to CXCL12 stimulation. In addition, CXCL12-dependent stimulation of CXCR4 on tumor cells has been shown to activate key downstream pathways including PI3K/AKT/mTOR and ERK1/2, which promote tumor cell survival proliferation, migration, and angiogenesis [29,33]. Similar to CXCR4, ACKR3 is also over expressed in a variety of solid malignancies including prostate and breast cancers and in each of these malignancies it has been shown to drive betta-arrestin-dependent tumor cell adhesion, invasion, survival and growth [34,35]. As a whole, it is therefore evident that, regardless of the exact mechanism leading to CXCL12/CXCR4/ACKR3 over-expression/stimulation in cancer, this axis plays multiple fundamental roles in cancer progression and as such, offers an important potential target for disease treatment.

## 5. Pathologic Role for CXCL12/CXCR4/ACKR3 in NSCLC

The majority of research regarding the role of CXCL12/CXCR4/ACKR3 in NSCLC has been focused on the CXCL12/CXCR4 pair. When considering the role of CXCL12/CXCR4 axis in NSCLC malignant propagation, three lines of research emerge as significant. The first line of research comes from publications that experimentally measured, in vitro and in vivo, the effects of CXCL12/CXCR4 interactions on NSCLC disease propagation. These studies have shown that in the tumor microenvironment, CXCL12/CXCR4 may foster local tumor growth and that high CXCR4 expressing tumor cells have a greater invasive and metastatic potential that may be blocked by CXCR4 antagonism [36,37,38,39,40,41]. These studies have also shown that CXCR4/CXCL12 interaction in the tumor microenvironment may help maintain an immune-quiescent microenvironment, and that antagonism of CXCR4 may render NSCLC cells more sensitive to chemotherapy [42,43]. More recently, an intriguing report by Tsai et al. has suggested that the EGFR-L858R mutation (which is a common driver of NSCLC) fosters the spread of lung adenocarcinoma cells to the pleural space via activation of the CXCL12/CXCR4 axis [44]. However, further attempts to replicate these findings by other groups have so far failed to validate this specific link between EGFR-L858R and CXCR4 [45]. With regard to ACKR3, studies have shown that over expression of ACKR3 promotes lung cancer growth and angiogenesis [34,46].

The second line of research comes from genetic-based retrospective studies that tested the association between single nucleotide polymorphism (SNP) in either CXCL12 or CXCR4 and the predisposition to develop NSCLC as well as the association of these SNPs with disease stage and prognosis. Two SNPs were tested: (a) the SNP in position 801 of the untranslated region of the CXCL12 gene–guanine to adenine (G→A) which has been shown to enhance CXCL12 expression and (b) the SNP at codon 138 of the CXCR4 gene-silent mutation cytosine to thymine (C→T)—of which the effect on CXCR4 expression is unknown [47,48]. The researchers found that individuals with the CXCL12 AA (high expressing genotype) and CXCR4 TT genotypes had higher odds ratios (an OR of 1.95, 95% CI 1.08–3.50, *p* = 0.018 and OR of 4.71, 95% CI 1.99–11.2, *p* < 0.0001, respectively) for lung cancer development. Furthermore, these genotypes were more frequently associated with advanced disease stage and with a worse prognosis [47,48]. Taken together, these results provide further evidence for a pro-malignant role of CXCL12/CXCR4 in NSCLC. 

The third line of research comes from studies that retrospectively evaluated the expression and tissue localization of CXCL12 and CXCR4 in NSCLC histopathological samples and correlated these findings with disease stage and long-term outcomes. Much work has been done in this field, with findings summarized in two recent meta-analyses. In brief, immunohistochemical staining for CXCR4 has demonstrated that strong expression of CXCR4 in the cytomembranous compartment of the lung cancer tumor cells correlated with a tendency of the tumors to locally invade neighboring anatomical structures and to also form distant metastasis at sites such as the brain and pleural space. Other reports have identified nuclear staining for CXCR4 in tumor cells and these findings correlated with improved prognosis, though when using different antibodies these results could not be replicated [37,38,39,41,49,50,51,52]. The expression and tissue localization of CXCL12 in NSCLC has also been studied. To illustrate, both our group and others have reported that the majority (80%) of NSCLC tumors expressed CXCL12 in the cytomembranous compartment and that high staining for CXCL12 correlated with an increased tendency for disease recurrence [42,52]. Other reports found linkage between high tumoral CXCL12 expression levels and both a higher T score and an increased tendency to form lymph node metastasis [39]. More recently, research has found that high CXCL12 expression in NSCLC tumor cells correlated with high staining of these cells also for phosphorylated CXCR4. In addition, high expression of CXCR4 in tumor cells correlated with lymph node metastasis and with high expression of CXCL12 in tumor-harboring lymph nodes. Together, these studies strongly suggest that tumor cells may indeed benefit from an autocrine CXCL12/CXCR4 loop in the tumor microenvironment and that high CXCL12 expression in lymph nodes may indeed promote the metastasis of CXCR4 expressing cells [52,53]. Finally, the two meta-analyses that have evaluated the overall effects of CXCL12/CXCR4 expression in NSCLC have reported that CXCR4 expression is closely associated with lymph node metastasis (OR = 3.79, 95% CI: 2.15–6.68), distant metastasis (OR = 3.67, 95% CI: 1.84–7.32), tumor stage (OR = 2.78, 95% CI: 1.77–4.39) and overall survival (HR = 1.63, 95% CI: 1.16–2.30). In addition, NSCLC patients with CXCR4 expression were more likely to be diagnosed with adenocarcinoma cancer (OR = 1.45, 95% CI = 1.07–1.95, *p* = 0.016), lymph node involvement (OR = 0.69, 95% CI = 0.50–0.96, *p* = 0.027), and distant metastasis (OR = 0.36, 95% CI = 0.14–0.93, *p* = 0.035). Both studies proposed that CXCR4 may be used as a new prognostic biomarker in NSCLC. They also suggested developing novel CXCR4 based diagnostic and therapeutic interventions for high CXCR4 expressing NSCLC tumors [54,55]. 

## 6. Diagnostic and Therapeutic Potential of CXCR4 Antagonists in NSCLC 

The accumulating knowledge regarding the fundamental roles that the CXCL12/CXCR4 plays in hematological and solid malignancies has driven multiple pharma companies to develop specific CXCR4 antagonists. These may be categorized into four major groups: Non peptide CXCR4 antagonists such as AMD3100, small peptide CXCR4 antagonists such as BL-8040, antibodies to CXCR4 such as LY2624587 and Ulocuplumab and modified agonist antagonists to CXCL12 such as NOX-A12 [56,57,58]. Reviewing the characteristics of and the therapeutic indications for which each of these inhibitors is being developed, is beyond the scope of this manuscript. Nonetheless, several general comments can be made: 

(1) CXCR4 inhibitors are known to induce profound mobilization of hematopoietic stem cells to the circulation. Consequently, in the setting of bone marrow transplantation, CXCR4 inhibitors are increasingly being utilized for stem cell harvesting. When considering the use of these agents for extended periods of time (in order to achieve prolonged and uniform inhibition of the CXCL12/CXCR4 axis), safety issues relating to prolonged mobilization of hematopoietic cells arise. Two clinical trials are currently looking into the safety of prolonged use (7 days) of CXCR4 inhibitors in solid malignancies [59] (NCT03277209 and NCT02179970): (1) The CXCR4 inhibiting effects (e.g., pharmacodynamics/affinity for CXCR /bystander effects) of each of the CXCR4 inhibitors that are being developed significantly differ from one another. Consequently, each inhibitor induces a unique response pattern. To illustrate, when the peptide CXCR4 antagonist LY2510924 was compared to the CXCR4 antibody LY2624587, the former induced sustained mobilization of hemtopoietic cells whereas the latter, barely induced any mobilization [60]. Similarly, in our own lab we have shown that the peptide CXCR4 antagonist BL8040 (previously named BKT-140) induces a more robust mobilization of hematopoietic cells as compared to AMD3100 [61]. Thus, when incorporating a CXCR4 inhibitor into a new therapeutic protocol, the characteristic response pattern to the specific inhibitor that is chosen must be thoroughly considered. 

(2) The vast majority of clinical trials with CXCR4 inhibitors in cancer have been performed in the field of hematological malignancies. In this field, CXCR4 inhibitors have shown encouraging results that prompted the initiation of several phase III clinical trials [59]. Remarkably, the success of CXCR4 inhibitors in the field of hematological malignancies is attributed in part to their potential to mobilize cancer cells into the circulation and thus render them more chemo-sensitive [59]. Undoubtedly, the chances of this mechanism playing a major role in solid cancer eradication is more questionable. 

(3) Only two clinical trials have evaluated the safety and effectiveness of CXCR4 inhibition together with standard chemotherapy for the treatment of solid malignancies. These trials were performed with the small peptide antagonist LY2510924 for the treatment of advanced SCLC and for the treatment of metastatic renal cell carcinoma. Both trials have shown that this approach is safe, and yet, neither has shown any clinical efficacy [62,63]. Multiple explanations may be derived from this observation. Nonetheless, a bold but reasonable assumption is that in these two specific scenarios, other pro-tumorigenic pathways may have been able to adequately compensate for the loss of CXCR4 signaling.

As a whole, it is important to recognize that sufficient evidence exists to support a pro-tumorigenic role for CXCL12/CXCR4 in NSCLC (acting locally to support tumor growth, to enhance angiogenesis, to promote chemo-resistance and to generate an immune permissive state as well as acting distally to promote metastasis formation). However, the challenge of developing CXCR4 therapeutics for lung cancer remains highly complex. 

Two promising directions that are being pursued in this regard are (1) the generation of radioactively-labeled small CXCR4 inhibitors for utilization in diagnostics and therapeutics of high CXCR4 expressing tumors [64,65], and (2) the usage of CXCR4 inhibitors to overcome tumor immune suppression in general and resistance to ICIs in particular [66,67,68,69]. With regards to the first direction, two groups have shown that labeling of the CXCR4 antagonist pentixafor with radioactive isotopes (68Ga and 64Cu) enabled the specific and accurate *in vivo* detection of high CXCR4 expressing tumors in the lung [64,65]. These findings are highly intriguing not only because they may, in the future, facilitate the selection of patients with high CXCR4 expressing tumors for anti-CXCR4 based therapeutics, but also because they open the way for labeling of CXCR4 antagonists with therapeutic radioactive isotopes such as 177Lu and 90Y for endoradiotherapy [70]. A major drawback of this approach, however, is the associated bone marrow toxicity that may emerge from such aggressive CXCR4-based ablative therapeutics [58]. 

With regard to the second direction of incorporation of CXCR4 antagonist into ICIs therapeutic protocols, several preclinical works have indicated that CXCL12/CXCR4 interactions in the tumor microenvironment mediate immune evasion and resistance to ICIs. In a preclinical pancreatic cancer model, it was shown that tumor associated fibroblast-derived CXCL12 mediated ICI resistance by excluding effector T cells from the tumor and that this effect was mitigated by AMD3100 leading to tumor eradication [66]. Further, in a colorectal-preclinical model, it was shown that resistance to antiangiogenic therapy was induced by the CXCL12/CXCR4 dependent recruitment of immunosuppressive Ly6C low monocytes to the TME and that this effect can be reversed by a CXCR4 blockade [69]. Similarly, in a hepatocellular carcinoma preclinical model, it was shown that CXCR4 inhibition in the TME was essential in order to facilitate response to ICIs following treatment with the anti-angiogenic broad kinase inhibitor sorafenib [67]. As a whole, these and other preclinical works, highlight that CXCL12/CXCR4 interactions—whether induced by hypoxia or by other mechanisms/cells in the TME—may foster the generation of an immune suppressive microenvironment that may significantly reduce the efficacy of ICIs. Following these works, much attention in the field of CXCR4 base therapeutics for solid malignancies has been shifted towards finding the right way to combine CXCR4 inhibitors with ICIs. Looking forward, multiple clinical trials are planned for various indications including pancreatic cancer, colorectal cancer and also metastatic NSCLC (NCT03337698) (NCT03168139) (NCT02907099). 

## 7. Concluding Remarks

Over the last decade, immune checkpoint inhibitors have revolutionized the field of cancer immunotherapy. In parallel, the field of CXCR4-based diagnostics and therapeutics for cancer have evolved significantly. Although much progress has been made with regard to hematological malignancies, solid cancers remain somewhat behind. Nevertheless, recent findings regarding the potential of CXCR4 antagonists to enhance the efficacy of other immune-activating drugs are fascinating as is the potential to utilize specific CXCR4 inhibitors for in vivo functional imaging of CXCR4 expression tumors. Given the important pathologic roles that CXCL12/CXCR4 axis play in NSCLC and given the urgent need for novel therapeutics for this devastating disease, it appears that now is the time to move forward and attempt to incorporate CXCR4 inhibitors in novel immune-based lung cancer therapeutic protocols. 
